# Non-canonical integration events in *Pichia pastoris* encountered during standard transformation analysed with genome sequencing

**DOI:** 10.1038/srep38952

**Published:** 2016-12-13

**Authors:** Jan-Philipp Schwarzhans, Daniel Wibberg, Anika Winkler, Tobias Luttermann, Jörn Kalinowski, Karl Friehs

**Affiliations:** 1 Fermentation Engineering, Bielefeld University, Universitätsstr. 25, Bielefeld, 33615, Germany; 2 Microbial Genomics and Biotechnology, Center for Biotechnology (CeBiTec), Bielefeld University, Universitätsstr. 27, Bielefeld, 33615, Germany; 3 Genome Research of Industrial Microorganisms, CeBiTec, Bielefeld University, Universitätsstr. 27, Bielefeld, 33615, Germany

## Abstract

The non-conventional yeast *Pichia pastoris* is a popular host for recombinant protein production in scientific research and industry. Typically, the expression cassette is integrated into the genome *via* homologous recombination. Due to unknown integration events, a large clonal variability is often encountered consisting of clones with different productivities as well as aberrant morphological or growth characteristics. In this study, we analysed several clones with abnormal colony morphology and discovered unpredicted integration events *via* whole genome sequencing. These include (i) the relocation of the locus targeted for replacement to another chromosome (ii) co-integration of DNA from the *E. coli* plasmid host and (iii) the disruption of untargeted genes affecting colony morphology. Most of these events have not been reported so far in literature and present challenges for genetic engineering approaches in this yeast. Especially, the presence and independent activity of *E. coli* DNA elements in *P. pastoris* is of concern. In our study, we provide a deeper insight into these events and their potential origins. Steps preventing or reducing the risk for these phenomena are proposed and will help scientists working on genetic engineering of *P. pastoris* or similar non-conventional yeast to better understand and control clonal variability.

The non-conventional yeast *Pichia pastoris* is a popular host for recombinant protein production, due to a highly efficient secretion mechanism and the possibility of reaching high product titres for simpler enzymes such as phytase and complex proteins containing multiple post-translational modifications, e.g. monoclonal antibodies^1^,^2^,^3^,^4^,^5^. Research by Kurtzman *et al*.^6^,^7^ resulted in the reclassification of the *P. pastoris* genus as *Komagatella*, including the subspecies *K. phaffii* and *K. pastoris*. However, they are still commonly referred to as *P. pastoris*. In recent years, the genetic toolbox for *P. pastoris* has been markedly expanded with several newly discovered native promoters, synthetic promoters and other regulatory elements^8^,^9^,^10^,^11^. The construction of optimized strains and vectors enabled new applications, e.g. the production of metabolites or expression of proteins lacking yeast specific hypermannosylation patterns^12^,^13^. Additionally, in a very recent publication Weninger *et al*.^14^ reported the first CRISPR/Cas9 system for *P. pastoris* opening up new possibilities for genetic engineering approaches.

Nevertheless, the most frequently used approach for introducing the target gene in *P. pastoris* is still the integration of an expression cassette into the genome *via* homologous recombination. The most popular target for integration is the *AOX1* (alcohol oxidase 1) locus that represents the stronger expressed of the two alcohol oxidases in *P. pastoris*. This approach usually involves the utilization of the *AOX1* promoter (p*AOX1*) as homologous sequence and as promoter of the target gene, because it offers very high expression levels and tight regulation^15^. After a successful integration, the gene expression can be induced with methanol. However, a clone with an intact *AOX1* can metabolize methanol at a higher rate, designated as “methanol utilization plus” (Mut^+^), complicating the maintenance of a constant induction^16^,^17^. Therefore, the application of expression cassettes with two homologous sequences targeted for mediating the replacement of *AOX1* is one possible technique. A knock-out mutation of *AOX1* leads to the phenotype “methanol utilization slow” (Mut^S^) easing process control and allowing to select for correct integration based on the phenotype.

Despite using comparatively long homologous sequences (ca. 1000 bp), a high variance in targeting efficiency is observed, indicating the prevalence of the non-homologous end joining (NHEJ) pathway in *P. pastoris*^18^,^19^,^20^. As a result, a high clonal variability is found after transformation, necessitating a time and labour-intensive screening process for the clone with the desired characteristics^21^,^22^. Concerning productivity characteristics, the different expression levels of clones typically originate from varying gene copy numbers^23^,^24^. The clonal variability is regarded as an inherent property of *P. pastoris* that is a by-product of the available and established transformation techniques in combination with the strong NHEJ pathway in this yeast species. Many other yeasts, filamentous fungi and higher eukaryotes that are used in biotechnological applications display similar or more pronounced clonal variabilities due to a predominant NHEJ pathway^25^,^26^,^27^, while in the model yeast *Saccharomyces cerevisiae* homologous recombination is dominant over the NHEJ pathway^28^. Different techniques to reduce the clonal variability in *P. pastoris* have been proposed^14^,^18^,^20^,^29^. However, disadvantages like high complexity, lower strain fitness or not yet fully realized methods for donor cassette integration have kept these techniques from replacing the established ones for the efficient construction of producer strains. Therefore, successive genetic manipulation steps, e.g. for the construction of biosynthetic pathways, have been comparatively challenging in *P. pastoris* and only a few applications have been reported so far^30^,^31^,^32^,^33^,^34^. To date, the humanisation of the N-glycosylation pathway in *P. pastoris* via multiple consecutive cloning steps has been the most sophisticated and successful genetic engineering endeavour^13^,^35^. Similar observations have been made for other non-conventional yeast like *Hansenula polymorpha, Kluyveromyces lactis* and *Yarrowia lipolytica*^25^,^36^,^37^.

In our previous study^24^, we concentrated on analysing the clonal variability found in 845 *P. pastoris* strains transformed with a GFP expression cassette targeted for *AOX1* replacement. By screening all clones for their Mut-phenotype, GFP productivity and gene copy number 31 clones with interesting features were selected for genome sequencing. The combination of experimental and genome data allowed the discovery of novel insights into the correlation between productivity and integration event. In the majority of cases variations in the productivity could be traced back to gene copy number related effects. No off-target integrations were found in clones with a high productivity, suggesting that the impact of such events on productivity was low.

During the Mut-phenotype assay, strains were discovered that displayed an abnormal colony morphology on plate. Some of these clones were also selected for genome sequencing. Here, we discuss the results for these strains, elucidating a variety of as yet unreported off-target integration events. Besides the disruption of random genes, the co-integration of DNA elements from the plasmid host *E. coli* KRX as well as the relocation of the *AOX1* locus to another chromosome were discovered. The detrimental effect of these events on the genetic integrity and strain morphology presents an additional burden for the screening process. These observations are especially relevant for metabolic engineering or knock-out experiments in *P. pastoris*, while the integration events detailed in our previous study^24^ are more pertinent to experiments targeting the creation of high producer strains.

In recent years, metabolic engineering of non-conventional yeast in general and *P. pastoris* in particular has gained more interest^11^,^38^. Therefore, the discussed integration events and the proposed theories explaining their origin will enable scientists working in this field to modify existing transformation and mutagenesis protocols in order to avoid these events. Based on this strategy, clonal diversity can be reduced and genetic engineering processes of higher complexity can be realized.

## Results

### Identification of clones with abnormal colony morphology

**Figure 1:**
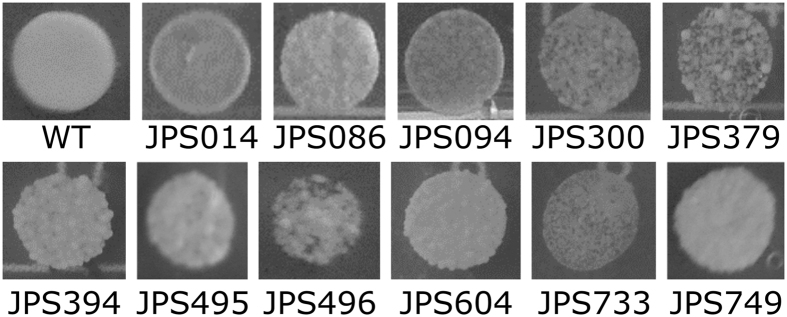
Colony morphology of wildtype *P. pastoris* CBS 7435 (WT) and irregular clones with crenulated morphology, grown on MD plates for 3 days at 28 °C. All mutant strains shown here were selected for genome sequencing.

During the plating assay for determination of the Mut phenotype multiple clones with a crenulated colony morphology were found (Fig. 1). The change in colony morphology indicated growth deficiencies, similar to the ones reported for *OCH1* knock-out strains in *P. pastoris* and other non-conventional yeast^20^,^39^,^40^. *OCH1* codes for a mannosyltransferase and its loss leads to a lower cell wall integrity, negatively affecting growth and temperature sensitivity as well as an increased flocculation^13^. It is also a popular knock-out target for humanizing the glycosylation pattern in *P. pastoris*^40^. Therefore, it was of interest to discover a potential genetic cause affecting the growth behaviour of clones with abnormal colony morphology. In total, 26 (=3%) of 845 analysed *P. pastoris* strains displayed a crenulated colony morphology and were divided into the group “Abnormal morphology”. Most of the clones of this group also belonged to other (productivity related) groups, mentioned in our previous report^24^. Table 1 summarizes the characteristics for all 26 clones with abnormal morphology. Except for the two strains JPS277 and JPS315, all clones had the Mut^+^-phenotype. Furthermore, only JPS094 and JPS280 displayed clearly elevated GFP expression levels of 2.6 and 3.5, respectively. Since most clones with an abnormal colony morphology had a gene copy number and GFP expression level of approximately 1, they would likely not be selected if looking for high producer strains. However, from a strain engineering perspective the integration events that lead to their change in morphology could be relevant and the strains were therefore further investigated. Eleven strains belonging to this group were sequenced and a variety of different integration events were discovered.

### Gene disruptions

**Figure 2:**
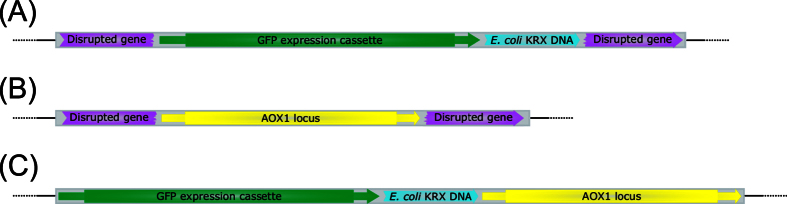
The different kinds of integration events discovered in sequenced *P. pastoris* clones with abnormal colony morphology. (**A**) Disruption of an untargeted gene by a GFP expression cassette fused to *E. coli* KRX DNA (**B**) Disruption of an untargeted gene by a re-integrated *AOX1* locus. (**C**) Co-integration of *E. coli* KRX DNA in fusion with an expression cassette at the *AOX1* locus.

In some cases, a gene disruption is the likely cause of the morphology change. For example, JPS496 (EMBL FBTT01000000) contained a disruption in the *MXR1* gene on chromosome 4, which codes for a methionine sulfoxide reductase (not to be confused with the similarly abbreviated methanol expression regulator 1 (*MXR1*)^41^). The gene product of *MXR1* in *S. cerevisiae* is part of the oxidative stress response and involved in the repair of damaged proteins by reducing methionine sulfoxide to methionine. Upon inactivation of *MXR1*, a change in colony morphology was noted in *S. cerevisiae*^42^. A disrupted *MXR1* in clone JPS496 could lead to the accumulation of damaged proteins in the cell and disrupt metabolic pathways, potentially explaining the observed morphology change. Interpretation of sequencing results for this clone were complicated by the fact that downstream of *MXR1* the paralogous gene *yahK* (alcohol dehydrogenase GroES domain protein) is located and also present on chromosome 3. Furthermore, *MXR1* itself has a paralogous copy on chromosome 3, although this copy is a fragment and only 567 bp long (*MXR1* on chromosome 4 is 669 bp long) and contains multiple mutations (>90 mismatches). In consequence, multiple possibilities existed to assemble the contigs related to the expression cassette and the *MXR1* locus in JPS496. To obtain a clearer picture, a PCR targeting the *MXR1* locus on chromosome 4 was performed, the primers are described in Table 2. The PCR assay indicated the integration of an >6 kb fragment in the *MXR1* locus on chromosome 4. Figure 2(A) shows the proposed integration event in JPS496. Accordingly, the integration occurred between the base pairs 40 and 41 of the *MXR1* gene. In frame transcription of the *MXR1* gene would thus continue in the newly integrated p*AOX1* and end after adding 10 amino acids (AA) at the stop codon TGA. Additionally, due to the integration between base pairs 40 and 41, a p. Lys14Arg mutation occurs. The resulting 23 AA gene product of the *MXR1*-p*AOX1*-hybrid is of different composition and misses the catalytic domain compared to the full length *MXR1* gene product (222 AA) (Fig. S1). It is therefore probably inactive. No significant homologies between the integrated DNA and *MXR1* exist, indicating that the insertion was likely the result of the NHEJ pathway. The effect of a *MXR1* deletion on the morphology of *P. pastoris* has not been reported yet.

A different kind of gene disruption was found in JPS014 (EMBL FBTG01000000). In this strain the *AOX1* locus relocated from its native site on chromosome 4 to chromosome 2 between base 2,062,817 and 2,062,818. As a result, gene PP7435_Chr2-1130 was disrupted (Fig. 2(B)). PP7435_Chr2-1130 is annotated as a hypothetical protein in *P. pastoris* CBS 7435^43^, but shows 100% identity to PAS_chr2-1_0178 in *P. pastoris* GS115^44^, which codes for a polyphosphatidylinositol (PtdIns) phosphatase. In JPS014 the in frame transcription of PP7435_Chr2-1130 would continue after the 781^st^ AA in the newly inserted p*AOX1*, adding 7 AA and end at a TGA stop codon. Furthermore, the insertion results in a c.G2346A silent mutation (p.Lys782Lys). In comparison to the full-length PP7435_Chr2-1130 protein (1069 AA), the gene product in JPS014 (788AA) is distinctly shorter and 79 AA of the predicted catalytic centre of the PtdIns phosphatase are mutated or missing (Fig. S2.). It is therefore likely, that the mutant protein in JPS014 is impaired in its activity. Due to the absence of sequence similarities between the disrupted gene and *AOX1*, the integration was likely caused by the NHEJ pathway. No knock-out study of this gene in *P. pastoris* was available at the time of writing the manuscript. However, it was shown that PtdIns phosphatases in *S. cerevisiae* are membrane bound proteins involved in cytoskeletal organization, signal transduction and membrane trafficking^45^. Stolz *et al*.^46^ could also observe morphological changes, namely increased cell wall thickness, of *S. cerevisiae* clones by performing knock-out experiments with different PtdIns phosphatases. Therefore, it is likely that the observed growth deficiency of strain JPS014 was caused by the disrupted PP7435_Chr2-1130 gene. PP7435_Chr2-1130 was disrupted by the *AOX1* region targeted for replacement by the GFP cassette, implying that *P. pastoris* used the excised *AOX1* locus (including the *AOX1* promoter and terminator) as a substrate for DNA repair. JPS014 displayed the Mut^+^-phenotype indicating the full functionality of the re-integrated *AOX1* locus.

**Figure 3:**
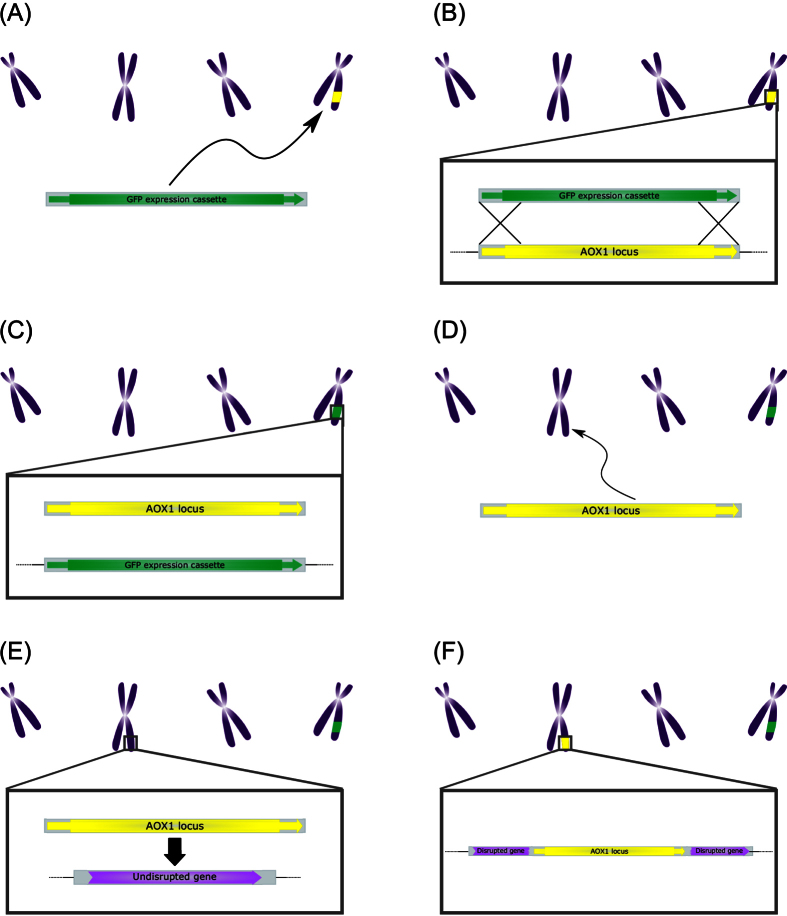
Proposed mechanism facilitating the relocation of the *AOX1* locus as found in clone JPS014. (**A**) After transformation the linear GFP expression cassette from pAHBgl-GFP moved to chromosome 4, where the *AOX1* locus is situated. (**B**) Two distal homologous sequences of the cassette with the chromosomal *AOX1* locus mediate a double cross-over recombination event (**C**) The cassette has replaced the *AOX1* locus on chromosome 4 (**D**) The excised linear *AOX1* locus moved to chromosome 2 (**E**) Potentially due to a DSB initiating the NHEJ pathway, the *AOX1* locus is integrated into a random gene on chromosome 2 (**F**) The untargeted gene PP7435_Chr2-1130 was disrupted. While the *AOX1* locus is still functional, the disruption of the untargeted gene likely led to the observed abnormal colony morphology in the case of JPS014.

Based on the assembled genome data, it was deduced, which chain of events could have led to the abnormal colony morphology observed for JPS014 (Fig. 3). Firstly, the native *AOX1* locus on chromosome 4 was replaced with the GFP expression cassette *via* the homologous double crossing over event, as intended. Secondly, the excised linear *AOX1* locus was not degraded, but instead managed to reach chromosome 2. Potentially, a double-strand break (DSB) in PP7435_Chr2-1130 induced the DSB repair pathway. The NHEJ pathway accepted the linear *AOX1* locus as substrate for the DSB repair, disrupting PP7435_Chr2-1130 in the process. In consequence, a *P. pastoris* clone was created with a single copy of the expression cassette at the desired locus, a functioning *AOX1* locus on chromosome 2 and a disrupted PP7435_Chr2-1130, likely causing the observed morphological changes.

It seems that even the successful replacement of the *AOX1* locus with an expression cassette can result in unforeseen and detrimental off-target effects. It is difficult to estimate, how frequently the excised *AOX1* locus was re-integrated, because in case of JPS014 it was only discovered due to morphological changes. Multiple other re-insertions of the *AOX1* locus with no or other gene disruptions could have occurred, but bypassed the selection process. If screening for deletion mutants in *P. pastoris*, it seems possible to miss successful recombination events due to the re-integration of the knock-out target at a different locus. Depending on the aim of the experiment, clones with interesting properties might be overlooked this way. It seems advisable to check for reintegration of the knock-out target at a different locus in knock-out experiments, in addition to confirming the removal of the target from its original site. Additionally, transformation strategies with only one homology sequence for integration should prevent the described event and ought to reduce the clonal variation caused by relocating knock-out targets.

### Co-integration of *E. coli* DNA

In four clones with irregular colony morphology (JPS086, JPS094, JPS300 and JPS496; EMBL FBTK01000000, FBTE01000000, FBTO01000000 and FBTT01000000, respectively) genome sequencing revealed the integration of *E. coli* KRX DNA elements directly adjacent to the GFP cassette (Fig. 2(C)). *E. coli* KRX was used as the plasmid host in all experiments. For a better understanding of the exact origin of these elements, we sequenced the *E. coli* KRX genome (unpublished). Using the genome data, the KRX DNA origin could be traced back to the F plasmid and the chromosome of its original host. In one *P. pastoris* clone a fusion of chromosomal and F plasmid DNA was discovered. Elements of *E. coli* KRX DNA present in strain JPS086, JPS094 and JPS300 contained multiple genes coding for proteins that are membrane associated in *E. coli*, identified *via* BLASTn. If not mentioned otherwise, KRX DNA elements in *P. pastoris* showed 100% identity to the chromosomal or F plasmid sequence of *E. coli* KRX. The ca. 4.6 kb of chromosomal *E. coli* KRX DNA in JPS086 contained genes coding for fimbria (*sfmF*) or that are involved in their expression and assembly (*sfmZ* and *smfH*). Adhesin associated genes (e.g. *ychA*) were encoded on the approx. 9.3 kb of F plasmid DNA integrated in JPS094. KRX F plasmid DNA (ca. 4.4 kb) found in clone JPS300 contained multiple membrane proteins or hypothetical proteins with assumed membrane association (*ybdA, yuaD, ybbA* and *ybaA*). In JPS496, a fusion of 553 bp chromosomal DNA and 947 bp F plasmid DNA from KRX was found. On the chromosomal portion, two sequences associated with transposases and on the F plasmid portion *finO* (fertility Inhibition) as well as a fragment of *traX* (acetylation of F-pilus) were identified. No genes coding for KRX proteins with (hypothetical) membrane association were detected in JPS496.

**Figure 4:**
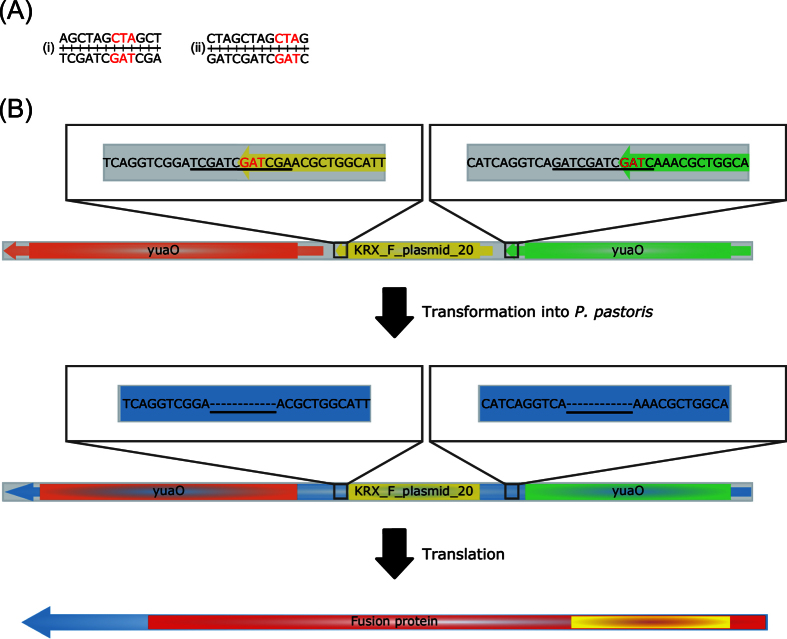
Observed modification of *E. coli* KRX DNA in *P. pastoris* clone JPS094. (**A**) Sequence of the two 12 bp long palindromic repeats found at the ends of (i) KRX_F_plasmid_20 and (ii) *yuaO*, respectively. The stop codons have been highlighted in red. (**B**) In its native form on the F plasmid of *E. coli* KRX both palindromic repeats (underlined) are present and contain a stop codon. After transformation into *P. pastoris* a deletion of both palindromic repeats was found in the genome assembly of JPS094. The loss of the stop codons indicated the formation of a ca. 1500 AA long fusion protein (blue), containing two conserved domains (red and yellow) after translation. The larger domain is associated with lipoproteins and autotransporters and the smaller one with outer membrane autotransporters.

Interestingly, the putative F plasmid DNA in clone JPS094 contained two 12 bp long deletions in comparison to the reference (Fig. 4). Both missing 12 bp sequences are palindromic, which indicates a common cause for their deletion. Potentially, the DNA repair mechanism that facilitated the integration of the pAHBgl-GFP and F plasmid hybrid in *P. pastoris* caused the deletions. It cannot be excluded that the excision already occurred in *E. coli* KRX. However, the presence of both 12 bp sequences in the KRX genome data contraindicates this theory. DNA repair mechanisms in yeast have problems with (long) palindromic sequences. In some cases, this phenomenon can lead to DSBs or inhibition of mismatch repair^47^,^48^. This fact suggests that the deletion occurred post-transformational in *P. pastoris* during or after integration of the foreign DNA. The deleted palindromic sequences lead to the excision of two stop-codons (of *yuaO* and KRX_F_plasmid_20) and the creation of a ca. 157 kDa large fusion protein. Even more surprisingly, the putative ORF ends 4 bp outside the integrated F plasmid DNA. In the adjacent *E. coli* backbone of pAHBgl-GFP, a TGA base-triplet serves as a presumed stop-codon. The DNA sequence of the resulting fusion protein shows similarity with *ycbB* (98% identity, BLASTn) of the *E. coli* ECC-1470 plasmid pECC-1470_100 (GenBank: CP010345) and the encoded protein has a high similarity (99% identity, BLASTp) to an outer membrane auto transporter barrel domain protein from *E. coli* OK1357 (GenBank: EFZ69416). In comparison to the auto transporter from *E. coli* OK1357, the putative fusion protein in JPS094 is 131 AA shorter. Using the NCBI Conserved Domain Database, two conserved domains associated with membrane anchoring and transport function (GenBank: PRK14849 and TIGR01414; BLASTx e-values 5.85 × 10^−33^ and 6.49 × 10^−23^, respectively) were found in the fusion protein^49^.

**Figure 5:**
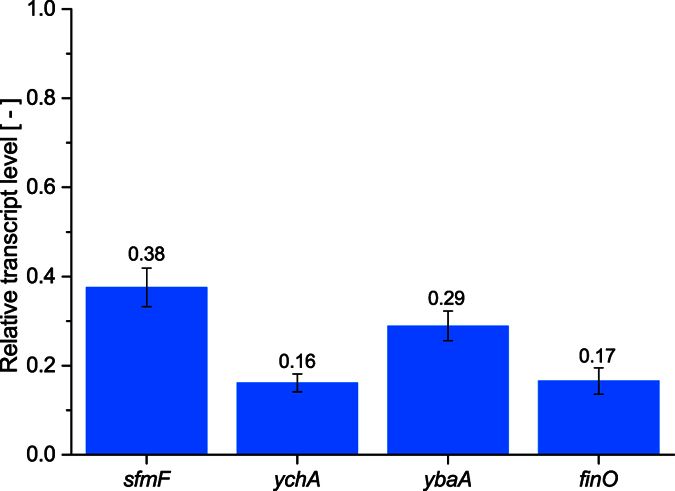
Relative transcriptional levels of *E. coli* KRX genes found in *P. pastoris* strains. *Sfmf* (fimbrial protein) in JPS086, *ychA* (adhesin *AidA* precursor) in JPS094, *ybaA* (signalling protein) in JPS300 and *finO* (fertility inhibitor) in JPS496. Samples for qRT-PCR experiments were taken in the exponential growth phase from cells grown in MD-medium. *ACT1* was used as housekeeping gene and values were normalized relative to the expression level of the *ACT1* gene in each sample. Error bars represent the standard deviation with n = 3.

With the exception of strain JPS496, a high abundance of genes coding for proteins that interact with the cell membrane were found on the integrated *E. coli* KRX DNA. This suggests that the observed morphological change have been the result of these proteins compromising the cellular integrity of *P. pastoris*. Since the irregular morphology was observed on both MD and MM plates, the expression of the *E. coli* proteins seemed to be independent of the methanol induction. In order to confirm the transcription of the *E. coli* fragments in the *P. pastoris* mutant strains, four representative targets were chosen for a qRT-PCR experiment. The targets were *sfmF* in JPS086, *ychA* in JPS094, *ybaA* in JPS300 and *finO* in JPS496. All four transcripts could be detected during the exponential growth phase in MD medium in their respective strains (Fig. 5). *sfmF* exhibited the highest and *ychA* the lowest relative transcript level compared to the endogenous control *ACT1*, with 0.38 and 0.16, respectively. At the same time, no expression of all four targets could be detected in *P. pastoris* CBS 7435 wild type. Interestingly, for most targets the Cq-value suggests that the transcript level was similar or higher in the mutant strains than in *E. coli* KRX (Fig. S3). It remains unclear how the transcription of the *E. coli* fragments is regulated in *P.pastoris*, but the results strongly suggest their activity independent of the methanol induction. Although the aberrant phenotypes indicate that the transcripts are also translated, further analysis *via* e.g. LC-MS would be needed to confirm the presence of the translated *E. coli* proteins in the mutant strains.

The morphological change of JPS496 was likely due to the disruption of *MXR1*, mentioned above. Other clones with irregular morphology were tested *via* PCR for the presence of the adhesin and fimbria genes seen in JPS094 and JPS086 *ychA* and *fimH*, respectively. It was found that three of them contained adhesin genes and six were positive for fimbria synthesis genes. Considering that these particular clones were only found to contain *E. coli* KRX DNA due to their abnormal colony morphology, the yet undiscovered presence of different elements from KRX in other strains appears possible. Taken together with the activity of these elements without methanol induction this could present multiple challenges for the biotechnological application of *P. pastoris*. Integration *via* the NHEJ pathway could lead to the disruption of untargeted genes. Gene products that interfere with the metabolism of *P. pastoris* could lead to decreased product yields or negatively affect growth behaviour. Further analysis on the activity of *E. coli* operons in *P. pastoris* is needed to clarify this theory. It has to be noted that no separate integration of KRX DNA was observed, it only occurred in fusion with an expression cassette. Therefore it seems, that *P. pastoris* efficiently degraded most foreign DNA and only the combination of KRX DNA and expression cassette facilitated an integration of *E. coli* DNA into the genome of *P. pastoris*.

**Figure 6:**
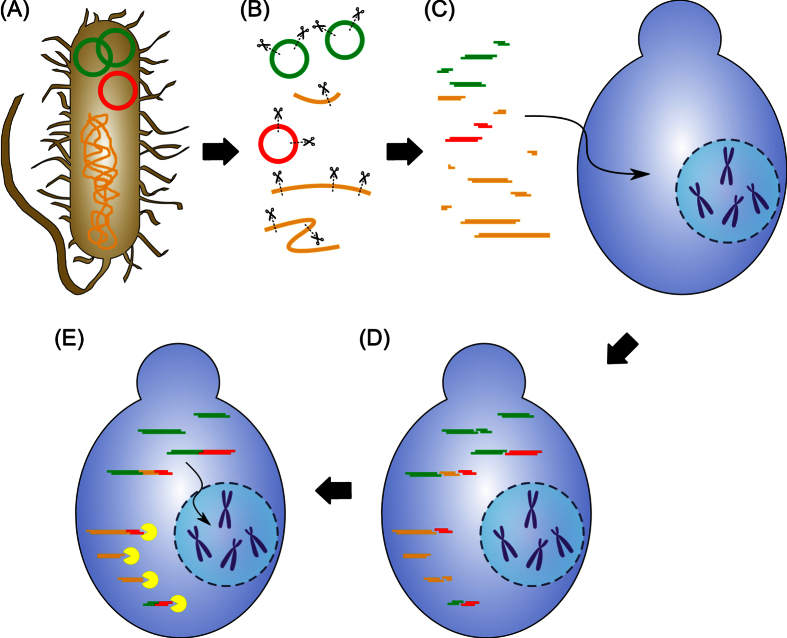
Schematic depiction of the proposed mechanism facilitating integration of *E. coli* host DNA in fusion with the expression cassette into the genome of *P. pastoris*. (**A**) *E. coli* cells containing chromosomal (orange) and F plasmid (red) DNA are used to produce the expression vector (green) (**B**) During plasmid isolation F plasmids and fragmented chromosomal DNA are co-extracted with the expression vector. Subsequent enzymatic digestion creates compatible sticky ends (**C**) Restricted DNA is transferred into *P. pastoris* cells via e.g. electroporation (**D**) *in vivo* various fragment combinations are ligated and form new hybrids (**E**) Expression cassettes and hybrids containing expression cassettes with homologies to chromosomal loci of *P. pastoris* are integrated into the genome, while non-homologous DNA is degraded.

Integrated *E. coli* KRX DNA was always directly adjacent to at least one *Bgl*II site on the chromosome or F plasmid of its original host. *Bgl*II was used for linearization of the expression cassette from pAHBgl-GFP, prior to transformation. Based on these results a theory regarding the mechanism underlying the observed phenomenon was formulated (Fig. 6). Presumably F plasmids and fragmented chromosomal DNA were co-extracted with the pAHBgl-GFP vector from *E. coli* KRX during plasmid preparation. *Via Bgl*II digestion the DNA was linearized, compatible sticky-ends were introduced and enabled the *in vivo* ligation of various fragment combinations in *P. pastoris* after transformation. *In vivo* ligation of linear DNA fragments in yeast has been reported before^50^,^51^. For *P. pastoris* it has been suspected that this mechanism plays a role in the generation of multi-copy clones^21^. The different combinations were then integrated into the genome of *P. pastoris* leading to the observed morphological changes. Combinations of ligated foreign DNA without homology sequence were likely degraded rather than integrated. While integration of such elements via the NHEJ pathway is possible, no integration events of that kind were found in any of the sequenced clones. Since the unwanted integration and activity of *E. coli* host DNA is not considered in commonly used *P. pastoris* transformation protocols, prevention mechanisms should be employed. *E. coli* strains containing F plasmids (like JM109, TOP10F’, XL10-Gold or DH5alphaF’) are often used for plasmid propagation in studies with *P. pastoris*^52^,^53^,^54^,^55^, but should be avoided. In order to prevent insertion of chromosomal DNA, either gel purification or PCR amplification of the expression cassette seems advisable. It has to be noted that in some cases the integrated *E. coli* host DNA fragments were of similar size as the expression cassette, reducing the potential effectiveness of gel purification. Furthermore, the use of blunt-end or rare cutters for expression cassette linearization ought to reduce the likelihood of hybrid formation.

## Discussion

Several non-canonical integration events were discovered in *P. pastoris* clones displaying a change in colony morphology. The disruption of genes analogous to *MXR1* and a PtdIns phosphatse from *S. cerevisiae* was shown to be the likely cause for growth deficiencies in two of the sequenced strains. In the case of the PtdIns phosphatase disruption, a relocation of the *AOX1* locus from chromosome 4 to chromosome 2 was observed. This event raises the question of how efficiently *P. pastoris* can re-integrate knock-out targets. The full functionality of the relocated *AOX1* locus urges scientists interested in efficient knock-out studies in *P. pastoris* (and other non-conventional yeast) to take similar events into consideration for the design of their experiments. Successful knock-outs are complicated, if the locus simply moves to a new site and clonal diversity is increased in case untargeted genes are disrupted by the relocated knock-out target. Novel techniques like the recently presented CRISPR/Cas9 system could help in knock-out studies^14^.

In multiple other strains morphological changes were possibly caused by the insertion and activity of DNA elements coding for membrane associated proteins from the plasmid propagation strain *E. coli* KRX. The methanol induction independent transcription of these *E. coli* elements in *P. pastoris* could be confirmed in a qRT-PCR assay. These findings strongly suggest that more care is needed, if transforming *P. pastoris*. Potentially the integration of other *E. coli* sequences remained undetected. Interestingly, in one case a presumed post-transformational modification of the KRX DNA was observed resulting in the excision of two stop codons and the creation of a triple-fusion protein with two membrane domains. The unwanted insertion of *E. coli* elements could cause multiple problems, e.g. in the generation of producer strains. Therefore, *E. coli* F^-^ strains should be used for studies involving *P. pastoris* transformation. Amplifying the expression cassette *via* PCR instead of using plasmid isolations should remediate the co-integration problem more effectively than gel purification. While gel purification of the expression cassette is a commonly used technique, we observed the co-integration of *E. coli* DNA elements of various sizes ranging from 1.5 to 9.3 kb. In some cases, these elements have a similar length in comparison to the expression cassette and thereby could by-pass a gel purification step. Nevertheless, PCR amplification could prove unsuitable in certain cases. Using blunt-end or rare cutters for plasmid linearization should also impede inadvertent co-integrations in these situations. Newly developed plasmids were recently published that offer sites for blunt-end linearization of an expression cassette targeting the *AOX1* locus for replacement^56^. While evolutionary horizontal gene transfer from bacteria to yeast (e.g. the acquisition of *URA1*^57^) was described previously, no co-integration of *E. coli* host DNA during genetic manipulation of *P. pastoris* has been reported so far.

Some of the sequenced strains (JPS379, JPS394, JPS495, JPS604 and JPS733) contained integration events that could not be related to the phenotype. They include the integration of a truncated expression cassette and mutations in *AOX1*. However, no gene disruptions were observed. While these events in some cases explained the low productivity, no clear correlation to the colony morphology could be deduced. In all analysed clones, no SNPs (single-nucleotide polymorphism) were detected that would explain the change in colony morphology.

In summary, the results elucidate the reason behind some of the causes for the clonal variability encountered in transformation experiments with *P. pastoris*. By focusing on strains with abnormal colony morphology, a group of clones undesirable for biotechnological applications can be better understood. Their change in morphology or growth behaviour is detrimental for production processes and in similar cases like Δ*OCH1* strains, special process strategies had to be developed to accommodate the changed physiology^58^. Additionally, the documented integration events and the proposed methods of preventing them enable scientists working with *P. pastoris* to devise optimized strategies for genetic engineering and mutagenesis of strains.

## Methods

### Strains and vector

Plasmids were constructed and propagated in *E. coli* KRX (Promega, Madison, WI; Genotype: [F´, traD36, ΔompP, proA + B+, lacIq, Δ(lacZ)M15] ΔompT, endA1, recA1, gyrA96 (Nalr), thi-1, hsdR17 (rk–, mk+), e14– (McrA–), relA1, supE44, Δ(lac-proAB), Δ(rhaBAD)::T7 RNA polymerase). Yeast experiments involved *P. pastoris* CBS 7435 (Δ*HIS4*) (Austrian Center of Industrial Biotechnology, Graz, Austria) and the wild type CBS 7435 (identical to NRRL Y-11430 and ATCC76273), which was obtained from the Spanish Type Culture Collection (Valencia, Spain) under the strain number CECT 11047. *P. pastoris* CBS 7435 (Δ*HIS4*) was transformed with *Bgl*II linearized expression cassette from pAHBgl-GFP according to Wu and Letchworth^59^. The composition and construction of pAHBgl-GFP was described previously^24^. In brief, a linearized GFP expression cassette (purified with Wizard^®^ Plus SV Minipreps DNA Purification System, Promega, Madison, WI) was transformed into *P. pastoris* and targeted the *AOX1* locus on chromosome 4 for replacement. Before transformation the linearized DNA was purified with the Wizard^®^ SV Gel and PCR Clean-Up System (Promega, Madison, WI).

### Cultivation conditions

*E. coli* KRX was cultivated in shake flasks at 37 °C with 120 rpm using Lysogeny Broth (LB) medium supplemented with 100 μg/mL Ampicillin.

*P. pastoris* was cultivated either in shake flasks, in 96-deep-well plates (Eppendorf, Germany) or on agar plates. Shake flask cultivations were performed with (Buffered) Minimal Dextrose ((B)MD, Invitrogen^60^), or Yeast Peptone Dextrose (YPD) medium and supplemented with 4 mg/L L-histidine when necessary. Deep-well plate experiments were performed according to Weis *et al*.^61^ and Hartner *et al*.^9^, with BMD and Buffered Minimal Methanol medium with 1 or 5 g/L methanol (BMM2 and BMM10, respectively). Agar plates contained Minimal Dextrose (MD) or Minimal Methanol (MM) medium^60^. All cultivations were carried out at 28 °C, with shake flasks being agitated at 120 rpm and deep-well plates at 340 rpm.

### Identification of Mut-phenotype and colony morphology

The Mut-phenotype was assayed based on the procedure described in the EasySelect™ Pichia Expression Kit^60^. Briefly, cells were grown in BMD medium for 60 hours, washed twice and then 5 μL aliquots of the resuspended cells were plated onto both MD and MM plates. After 2–3 days of incubation at 28 °C, the Mut-phenotype could be determined based on the growth behaviour. Additionally, the plating test allowed the visual identification of clones with irregular colony morphology.

### Isolation of genomic DNA and PCR assays

For the extraction of genomic DNA (gDNA) from *P. pastoris* the MasterPure™ Yeast DNA Purification Kit from Epicentre (Madison, WI) was used according to the manufacturer’s protocol.

gDNA from relevant clones was used for different PCR assays. The sequence of the primers used in these assays can be found in Table 2.

### Genome sequencing and bioinformatic analysis of *P. pastoris* strains

The performed procedures for *P. pastoris* clones selected for genome sequencing were recently reported^24^. In brief, the gDNA quantity and quality was assayed *via* the Quant-iT PicoGreen dsDNA kit (Invitrogen, Waltham, MA) and gel-electrophoresis, respectively. gDNA of sufficient quality was sequenced on an Illumina MiSeq system using a paired-end library prepared with the TruSeq sample preparation kit (Illumina, San Diego, CA). The raw data was assembled *de novo* in the GS *De Novo* Assembler (Version 2.8, Roche, Basel, Switzerland) with default settings. All assembled genomes can be found under the study id **PRJEB12220** in the EBI database.

Bioinformatic analysis involved similarity analysis using the BLASTn algorithm^62^ and a local database including the pAHBgl-GFP vector sequence. Hits with an e-value >1 × 10^−20^ and a sequence identity of 100% were analysed in more detail. Gaps in the vector sequence were closed in an *in silico* approach applying CONSED^43^,^63^,^64^. This approach also allowed the identification of insertion sites of the expression cassette into the genome of *P. pastoris*.

### Genome sequencing, annotation and sequence comparison of the *E. coli* KRX genome

DNA was extracted using the Wizard^®^ Genomic DNA Purification KIT (Promega, Madison, WI). *E. coli* KRX genome sequencing and bioinformatic analysis was performed analogous to the method described for *P. pastoris*, resulting in the draft sequence of the chromosome and the F plasmid (unpublished). *E. coli* KRX contigs were compared to the *P. pastoris* draft genomes sequences using BLASTn^62^. Hits with an e-value >1 × 10^−20^ and a sequence identity of 95% were analysed in detail to detect horizontal gene transfer between the *P. pastoris* strains and *E. coli* KRX chromosomal or F plasmid DNA.

### qRT-PCR experiments

*E. coli* KRX, *P. pastoris* CBS 7435 and the mutant strains JPS086, JPS094, JPS300 and JPS496 were grown as described above in shake flasks containing LB-Medium or MD-Medium, respectively. Samples taken in the exponential growth phase were employed for RNA isolation using the RNeasy Mini Kit (Qiagen, Hilden, Germany), with both an on- and off-column gDNA digestion step. RNA quantity and the absence of gDNA and other contaminants was assayed with the DropSense16 system (Trinean, Ghent, Belgium). Four different primer sets targeting the various *E. coli* fragments found in the sequenced *P. pastoris* mutant strains were designed using the NCBI Primer-BLAST tool, making sure no off-target activity on either the *E. coli* or *P. pastoris* genome was found. Additionally, the primer pair for quantification of the housekeeping gene *ACT1* (β-actin) in *P. pastoris* was taken from literature^65^. All primers exhibited similar T_M_-values (59 ± 2 °C) and amplicon sizes (170 ± 19 bp). The sequences and amplicon sizes of all employed primer pairs can be found in Table 2. Per reaction 50 ng RNA free of gDNA was used as template. Employing the SensiFAST^TM^ SYBR^®^ No-ROX One-Step Kit by Bioline (London, UK), samples were measured on the LightCycler^®^ 96 system (Roche, Basel, Switzerland) in biological triplicates with technical duplicates. For *P. pastoris* samples the transcript level of *E. coli* genes was normalized against the *ACT1* level of the same strain via the 2^−ΔΔCt^ method^66^. After 40 cycles the specificity of the amplicons was confirmed via a melting curve.

## Additional Information

**How to cite this article**: Schwarzhans, J.-P. *et al*. Non-canonical integration events in *Pichia pastoris* encountered during standard transformation analysed with genome sequencing. *Sci. Rep.* **6**, 38952; doi: 10.1038/srep38952 (2016).

**Publisher’s note:** Springer Nature remains neutral with regard to jurisdictional claims in published maps and institutional affiliations.

## Supplementary Material

Supplementary Information

## Figures and Tables

**Table 1:** Characteristics of P. pastoris strains displaying an abnormal colony morphology, determined as detailed in Schwarzhans *et al*. 24.

Strain	Mut-Phenotype	GCN	ExpLvl
JPS014	Mut^+^	0.7	0.3
JPS086	Mut^+^	0.4	1.1
JPS094	Mut^+^	2.5	2.6
JPS165	Mut^+^	0.9	1.0
JPS247	Mut^+^	0.9	0.6
JPS277	Mut^S^	0.9	0.9
JPS280	Mut^+^	2.4	3.5
JPS300	Mut^+^	0.5	0.5
JPS315	Mut^S^	0.9	0.8
JPS336	Mut^+^	1.0	0.1
JPS347	Mut^+^	0.3	1.1
JPS379	Mut^+^	0.1	0.0
JPS386	Mut^+^	5.2	1.2
JPS394	Mut^+^	0.6	0.8
JPS495	Mut^+^	0.1	1.7
JPS496	Mut^+^	0.5	0.5
JPS581	Mut^+^	0.8	1.4
JPS603	Mut^+^	3.1	0.6
JPS604	Mut^+^	0.2	0.0
JPS626	Mut^+^	0.6	0.7
JPS636	Mut^+^	0.8	1.0
JPS733	Mut^+^	0.6	0.3
JPS749	Mut^+^	0.7	0.4
JPS768	Mut^+^	0.6	0.5
JPS804	Mut^+^	0.7	0.6
JPS835	Mut^+^	0.8	0.8

A gene copy number (GCN) <1 can result from e.g. a reduced PCR efficiency during qPCR, but is still indicative of a single copy of the GFP cassette. The GFP expression level (ExpLvl) was normalized against a reference Mut^S^ strain with one copy of the cassette. Underlined strains were selected for genome sequencing.

**Table 2:** Primers used for PCR assays and qRT-PCR.

Primer name	Sequence	Purpose
MXR1-FW	GAGAAGGATAGGATCACGGTGGCC	*MXR1* locus amplification
MXR1-RV	GCGCCAGAGACATTCTACAGGGAG	
ADH-FW	CCCTTTATCACCAACTTGAACTCTCACATTCCCCC	Amplification of Adhesin *AidA* precursor *ychA* found in JPS094
ADH-RV	GGCCTGGTGCAACAGGATGGATTAATATTTTTAATGG	
FIM-FW	CTCAAATATCTTTAATAGCACCAATAACCAGCCAGGG	Amplification of Adhesin fragment *fimH* and Fimbrial protein found in JPS086
FIM-RV	CATCTGCCTAAATCTACCGTCTTATCAATATCCGC	
ACT1-FW	GGTGTGGTGCCAGATCTTTT	qRT-PCR; Housekeeping gene *ACT1* in *P. pastoris*; Amplicon = 181 bp
ACT1-RV	AGTGTTCCCATCGGTCGTAG	
sfmF-FW	TACTGAACGCTGGCGATACC	qRT-PCR; *sfmF* in JPS086 and *E. coli* KRX, Amplicon = 163 bp
sfmF-RV	AATTCGATGGCGACGGTTTG	
ychA-FW	GCAGCGTGCCATTATTACCG	qRT-PCR; *ychA* in JPS094 and *E. coli* KRX, Amplicon = 189 bp
ychA-RV	TTGGGCAATCTGATGCACCT	
ybaA-FW	TCGGAACGGAACGGATAAGAC	qRT-PCR; *ybaA* in JPS300 and *E. coli* KRX, Amplicon = 160 bp
ybaA-RV	TGCAAGTGTGAAAGCTCAGCA	
finO-FW	AATGTCACCACGCCACCAAA	qRT-PCR; *finO* in JPS496 and *E. coli* KRX, Amplicon = 159 bp
finO-RV	CTTCAGGGTGTTCACGGCAT	
