# Supplementary material for: Non-canonical integration events in Pichia pastoris encountered during standard transformation analysed with genome sequencing
Source: Sci Rep. 2016 Dec 13;6:38952. doi: 10.1038/srep38952 (PMC5154183; doi:10.1038/srep38952)
Supplement: Supplementary Information [file srep38952-s1.pdf]

Supplementary information

Non-canonical integration events in *Pichia pastoris* encountered during standard transformation analysed with genome sequencing

Jan-Philipp Schwarzhans[1,2], Daniel Wibberg[3], Anika Winkler[2], Tobias Luttermann[1], Jörn Kalinowski[2], Karl Friehs[1,*]

[1] Fermentation Engineering, Bielefeld University, Universitätsstr. 25, Bielefeld, 33615, Germany

[2] Microbial Genomics and Biotechnology, Center for Biotechnology (CeBiTec), Bielefeld University, Universitätsstr. 27, Bielefeld, 33615, Germany

[3] Genome Research of Industrial Microorganisms, CeBiTec, Bielefeld University, Universitätsstr. 27, Bielefeld, 33615, Germany

*Corresponding author at: karl.friehs@uni-bielefeld.de

```
MXR1              1     MKGYIFLLAPSSSKASGFAFMGSFRNFFKRSSSHMPVNSSLVSSNIRKSPQDKVVTVAGGCFWGLEHIYKMHFKDRIVDT   80
JPS496 Mutant     1     MKGYIFLLAPSSSRSNIQRRKVE*--------------------------------------------------------   24
MXR1              81    QVGFANGNVANPTYTQICLGMTHHAEVLQISYNPDVTSFNELIDFFFLVHDPTQKDRQGNDIGSQYRSAVFYLDEKEKEI   160
JPS496 Mutant           --------------------------------------------------------------------------------
MXR1              161   TEQSLAEAQKKWFPHHKIVTQVEKLTSYLDADDYHQDYLMKNPNGYHCPTHVLRKEPKSMSI*                   223
JPS496 Mutant           ---------------------------------------------------------------
```

Figure S1: Alignment of the AA sequence of the native *mxr1* protein (GenBank: CCA41165.1) from *P. pastoris* CBS 7435 and the mutant found in strain JPS496, based on the coding sequence found during genome sequencing. Mutations are highlighted in red. The alignment was performed using the Constraint-based Multiple Alignment tool (Cobalt) of the NCBI. Via the NCBI Conserved Domain Database a peptide methionine sulfoxide reductase domain (underlined) was detected in the region AA 56-203 of the native protein.

```
PP7435_CHR2-1130  1    MRVLIHEKTRLIALVSSTHCLLIKYIKDKNQCAVDFVEKESFSPKHMKPLSRNKAHGFLGLINVNEDVFLCVITGKTEVA  80
JPS014 Mutant     1    MRVLIHEKTRLIALVSSTHCLLIKYIKDKNQCAVDFVEKESFSPKHMKPLSRNKAHGFLGLINVNEDVFLCVITGKTEVA  80
PP7435_CHR2-1130  81   TPTDGETVNKIYNVEFHCLNRDAWDFLELDGNGYPITTSDVNEEALRSRGSTEASDNHLSYEQNPTYELRRLLSNGSFYY  160
JPS014 Mutant     81   TPTDGETVNKIYNVEFHCLNRDAWDFLELDGNGYPITTSDVNEEALRSRGSTEASDNHLSYEQNPTYELRRLLSNGSFYY  160
PP7435_CHR2-1130  161  STNFDLTSTLQSRDVNSDSLSFDSFHLDYMWNSYMMKEVVNFRDRLPTDSKKILDRNGFLTTVIRGFAETFRTRIGHQKC  240
JPS014 Mutant     161  STNFDLTSTLQSRDVNSDSLSFDSFHLDYMWNSYMMKEVVNFRDRLPTDSKKILDRNGFLTTVIRGFAETFRTRIGHQKC  240
PP7435_CHR2-1130  241  NATIISKQSWKRAGTRYNARGIDDEGYVANFVETELILHSKDFIYAYTEVRGSVPIFWEQDTALVNPKVTITRSLEATEP  320
JPS014 Mutant     241  NATIISKQSWKRAGTRYNARGIDDEGYVANFVETELILHSKDFIYAYTEVRGSVPIFWEQDTALVNPKVTITRSLEATEP  320
PP7435_CHR2-1130  321  VFEKHFAALNGKYGPVHIVNLLSTKPSEIGLSNTYRKHFEIVNKKGSPQAYLTEFDFHKETGKNYALATKVIPFLEESIY  400
JPS014 Mutant     321  VFEKHFAALNGKYGPVHIVNLLSTKPSEIGLSNTYRKHFEIVNKKGSPQAYLTEFDFHKETGKNYALATKVIPFLEESIY  400
PP7435_CHR2-1130  401  DFDYFSYDVKNQKVLTLQKGVFRTNCLDCLDRTNVVQQVISNATLNMFLQRHNLNTNYDSDLFNKHNTLWADHGDAISQI  480
JPS014 Mutant     401  DFDYFSYDVKNQKVLTLQKGVFRTNCLDCLDRTNVVQQVISNATLNMFLQRHNLNTNYDSDLFNKHNTLWADHGDAISQI  480
PP7435_CHR2-1130  481  YTGTNALKSSFTRSGKMGIAGALSDATKSISRMYINNFQDKAKQVTIDTLLGKMSNQVEVRIFDPVSDHVDNELSKLKSQ  560
JPS014 Mutant     481  YTGTNALKSSFTRSGKMGIAGALSDATKSISRMYINNFQDKAKQVTIDTLLGKMSNQVEVRIFDPVSDHVDNELSKLKSQ  560
PP7435_CHR2-1130  561  FSAEDDIRIFTGSYNLGGTAYADDFTDWLFPKENGGIEGAPDVVILGFQEVVELTASNILNSDSSRSHYWSEEIKTQLNK  640
JPS014 Mutant     561  FSAEDDIRIFTGSYNLGGTAYADDFTDWLFPKENGGIEGAPDVVILGFQEVVELTASNILNSDSSRSHYWSEEIKTQLNK  640
PP7435_CHR2-1130  641  ISSSKYILLRSEQMTSLLLLFFIKEDKMPKVTQVEGCSKKTGLGGITANKGAVALRFSFGSTTFCLLNSHLAAGLNSVVE  720
JPS014 Mutant     641  ISSSKYILLRSEQMTSLLLLFFIKEDKMPKVTQVEGCSKKTGLGGITANKGAVALRFSFGSTTFCLLNSHLAAGLNSVVE  720
PP7435_CHR2-1130  721  RNNDFTTISQGIRFSRNKTIYDHDCVIWLGDLNYRVPLPNELVRSSALNGVYDELLAEDQLKTEMVHKGAFADFYEMKIN  800
JPS014 Mutant     721  RNNDFTTISQGIRFSRNKTIYDHDCVIWLGDLNYRVPLPNELVRSSALNGVYDELLAEDQLKHPKTKG*-----------  789
PP7435_CHR2-1130  801  FLPTYKYDKGTSVFDTSEKQRVPSWTDRILYRGKRLQQVNYNSVQSITISDHKPIYGTFKAHVTYVDERTKLTLMRKIYD  880
JPS014 Mutant          --------------------------------------------------------------------------------
```

```
PP7435_CHR2-1130  881   DYRRDPKSEASSASSNGDKFGDLIDFNDASSSSNTSIYTETEGKQVSQPVPGHFPPPPPPPRNRVTPPQPSTPETPPPPG   960

JPS014 Mutant           --------------------------------------------------------------------------------

PP7435_CHR2-1130  961   YALNMAPLIPSRANSGNNTRPSSPFEVSTDRAKAEISTKPVRPMVPDKPSSLKAMSPENTIQTVPPVQKAISTDSLNTAS   1040

JPS014 Mutant           --------------------------------------------------------------------------------

PP7435_CHR2-1130  1041  AAPPPPPPPRKQESINSSMSAFPVLMPKKK*                                                   1070

JPS014 Mutant           -------------------------------
```

Figure S2: Alignment of the AA sequence of the native PP7435_Chr2-1130 protein (GenBank CCA38807.1) from *P. pastoris* CBS 7435 and the mutant found in strain JPS014, based on the coding sequence found during genome sequencing. Mutations are highlighted in red, with the silent mutation c.G2346A in green. The alignment was performed via Cobalt of the NCBI. Two domains (underlined) were predicted in the native protein, using the NCBI Conserved Domain Database: a *Sac*I homology domain (AA 56-381) and a catalytic inositol polyphosphate 5-phosphatase (INPP5c) domain (AA 567-861).

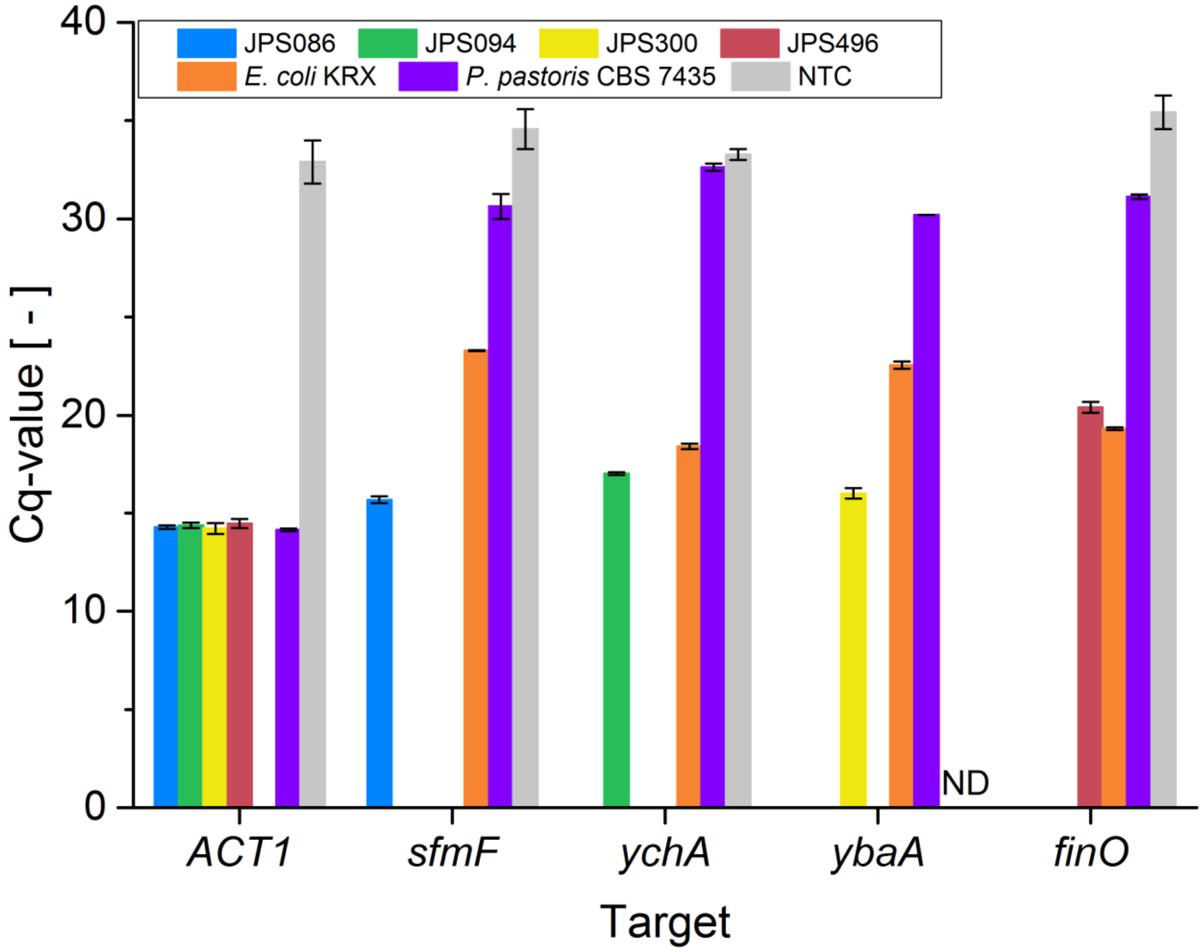

Figure S3: Cq-values measured during qRT-PCR experiments for the different target amplicons in *E. coli* KRX, *P. pastoris* CBS 7435 and the mutant strains JPS086, JPS094, JPS300 and JPS496. Additionally, the no template control (NTC) is shown. The targets are the housekeeping gene *ACT1* of *P. pastoris* as well as the *E. coli* genes *sfmF* (fimbrial protein), *ychA* (adhesin *AidA* precursor), *ybaA* (signaling protein) and *finO* (fertility inhibition). For the target *ybaA* no detectable (ND) signal was found in the NTC after 40 cycles. The melting curve analysis after 40 cycles revealed that in the *P. pastoris* CBS 7435 samples secondary amplicons were the cause for Cq-values < 33.3. Error bars indicate the standard deviation with n = 3.
